# Recognition memory-induced gene expression in the perirhinal cortex: A transcriptomic analysis

**DOI:** 10.1016/j.bbr.2017.04.007

**Published:** 2017-06-15

**Authors:** Hannah Scott, Mark F. Rogers, Helen L. Scott, Colin Campbell, Elizabeth C. Warburton, James B. Uney

**Affiliations:** a School of Physiology and Pharmacology, University of Bristol BS8 1TD, UK; b School of Clinical Sciences, University of Bristol BS8 1TD, UK; c Intelligent Systems Laboratory, Department of Engineering Mathematics, University of Bristol BS8 1TD, UK

**Keywords:** Memory, Perirhinal cortex, RNA sequencing, Transcription factor, Alternative splicing, Extracellular matrix

## Abstract

We have used transcriptome analysis to identify genes and pathways that are activated during recognition memory formation in the perirhinal cortex. Rats were exposed to objects either repeatedly, so that the objects become familiar, or to novel objects in a bow-tie maze over six consecutive days. On the final day, one hour after the last exposure to the series of objects, RNA from the perirhinal cortex was sequenced to compare the transcriptome of naïve control rats and rats exposed to either novel or familiar stimuli. Differentially expressed genes were identified between group Novel and group Familiar rats. These included genes coding for transcription factors, GDNF receptors and extracellular matrix-related proteins. Moreover, differences in alternative splicing were also detected between the two groups, which suggests that this post-transcriptional mechanism may play a role in the consolidation of object recognition memory. To conclude, this study shows that RNA sequencing can be used as a tool to identify differences in gene expression in behaving animals undergoing the same task but encountering different exposures.

## Highlights

• The transcriptome was characterised following exposure to novel or familiar objects.
• Differential expression of transcription factors and GDNF receptors was observed.
• Genes coding for extracellular matrix proteins are differentially expressed.
• Differences in alternative splicing were detected.

## 1 Introduction

Recognition memory allows us to make judgements regarding whether we have encountered something before, and hence to discriminate a novel from a familiar stimulus. A number of studies in both humans and animals have shown that such familiarity discrimination depends on the integrity of the perirhinal cortex in the medial temporal lobe [1], [2], [3]. It has been shown that protein synthesis in the perirhinal cortex is necessary for the consolidation of long-term recognition memory [4]. Furthermore, several immediate early genes, e.g. *c-Fos* and transcription factors, which are increased during recognition memory are suggested to have a causal role [5], [6], [7], [8], [9], [10], [11]. For example, a repeated observation is an increase in the number of c-Fos positive neurons in the perirhinal cortex in response to novelty [5], [11], [12], [13]. Furthermore, levels of the transcription factor cAMP response element-binding protein (CREB), itself an activator of immediate early genes, were found to be increased in the perirhinal cortex of rats exposed to novel compared to familiar objects [10]. Other immediate early genes functionally associated with recognition memory include early growth response 1 (Egr1; also known as Zif268) [7], activity-regulated cytoskeleton-associated protein (Arc) [6] and nuclear receptor 4a family member NR4a2 [8]. The observation that transcription factors are required in recognition memory suggests that gene expression may be an important process underlying long-term recognition memory formation.

To better understand the changes in gene expression that forge recognition memory, we have profiled the transcriptome of the perirhinal cortex following a recognition memory task. Using RNA sequencing we aimed to investigate the differences in gene expression between rats that encountered novel objects and those that saw highly familiar objects. The animals were exposed to the objects using a bow-tie maze [14], which allows recognition to be tested over multiple trials, and previous studies have shown that this protocol results in differential *c-Fos* expression selectively in the caudal perirhinal cortex, in response to exploration of novel and familiar objects [14].

In addition to the exploratory nature of this study, we aimed to investigate specific gene groups and pathways that have been associated with recognition memory. Firstly, due to the previously observed association of transcription factors with exposure to novelty, we predicted an increase in the expression of transcription factors and immediate early genes in the perirhinal cortex of group Novel compared to Control or Familiar. Secondly, signalling pathways such as the neurotrophic signalling pathway have been shown to be important in the first two hours following exposure to novel objects [15], [16], [17], [18]. Therefore we hypothesised that an upregulation of transcripts coding for neurotrophic factors and receptors might occur in group Novel. Thirdly, we explored the potential role of alternative splicing in the perirhinal cortex.

## 2 Materials and methods

### 2.1 Animals

Male Lister Hooded rats (∼350–450 g; Harlan Laboratories, UK) were used for all experiments. Rats were kept on a reversed 12-h light/dark cycle (lights on 20.00-08.00 h) and all behavioural testing took place during the dark phase. Prior to the start of the behavioural experiments, the animals were placed on food restriction with daily access to food for a period of 2 h. The rats were kept at over 85% of their free-feeding weight. Water was available without restriction.

All animal procedures were conducted in accordance with the United Kingdom Animals Scientific Procedures Act (1986) and associated guidelines. All efforts were made to minimise any suffering and the number of animals used.

### 2.2 Behavioural task

**Fig. 1:**
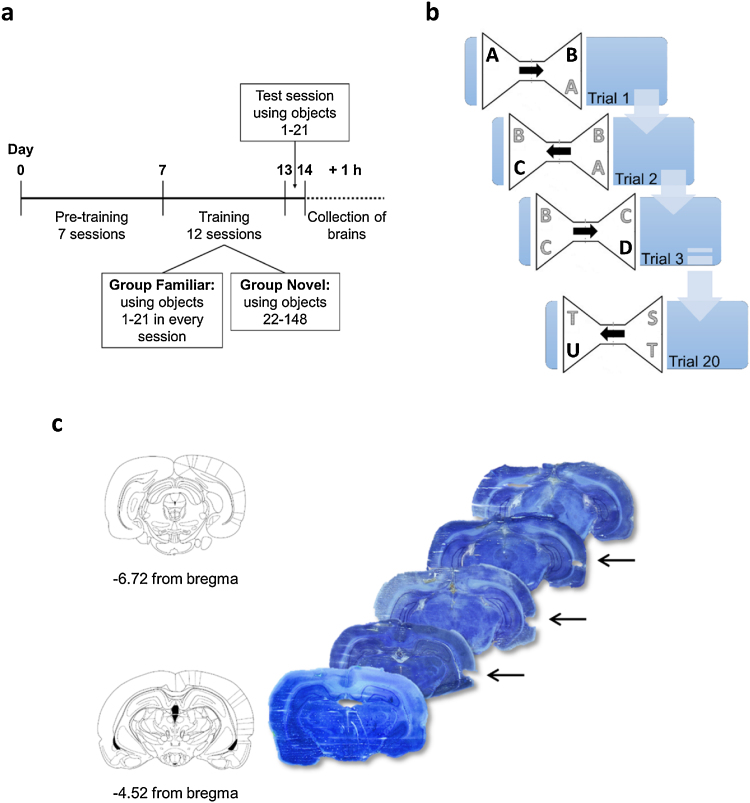
Experimental set-up. **a**. Timeline of the bow-tie maze experimental set-up. **b**. Schematic of the first three and the last of 20 trials of the bow-tie maze training session. Objects labelled in black are novel (for this session) and those in grey are familiar as they have been explored on the previous trial. Arrows indicate the movement of the rat. Each trial lasts 1 min **c**. Rat brain sections showing perirhinal cortex regions (arrows) extracted between −4.6 and −6.7 mm from bregma. Selected sections and map slices before and after samples were taken are shown.

The bow-tie maze task [14] consisted of two phases − pre-training and training – followed by the test session (Fig. 1a). The rats were divided into three groups – group Familiar, group Novel and group Control. Rats were housed so that one rat from group Familiar and one from group Novel shared a cage to ensure the rats were treated similarly across groups. Control rats were also food restricted and handled daily throughout the procedure but they were not subjected to any behavioural training or testing.

#### 2.2.1 Apparatus

Testing took place in a bow-tie shaped maze consisting of a grey wooden floor and metal walls. A guillotine door that could be operated manually separated the two triangular sides of the maze. At each end of the maze a food well was attached centrally to the floor, which could be baited with a food reward in the form of a sucrose pellet (OmniTreat™ 45 mg tablets; TestDiet, Sandown Scientific, Hampton, UK). Objects were placed into the left and right corners at either end of the maze. This set-up ensured that neither of the objects was associated with the food reward. The objects were junk objects, including toys, household items and decorative objects, in varied colours, shapes, materials and sizes. In between behavioural sessions, the objects were cleaned with 100% ethanol to eliminate olfactory cues. The animals’ behaviour was recorded via an overhead camera.

#### 2.2.2 Pre-training

In order for the rats to learn to shuttle from one end of the maze to the other when the sliding door was opened and to collect a food reward, the animals were subjected to daily training for 7 days. On day 1 the rats were allowed to explore the arena (without the guillotine door in place) in pairs for 30 min. Sucrose pellets were distributed across the maze and in the food wells. On days 2 and 3, the rats were allowed to explore the maze individually for 20 min. Only the food wells contained reward pellets (one pellet at a time) and they were constantly re-baited to encourage shuttling from one side of the arena to the other. Days 4 and 5 were the same except that the central guillotine door was used to control the movement of the rat. On days 6 and 7 three different pairs of objects were introduced. These were not used in the training phase.

#### 2.2.3 Training

The training phase consisted of two training sessions per day, one in the morning and one in the afternoon, over 6 consecutive days (training sessions 1–12). The set-up of the training sessions is displayed in Fig. 1b. At the start of each training session the rat, from group Novel or group Familiar, was placed into one side of the arena with the guillotine door shut, where it encountered one object A which it had the opportunity to explore for 1 min. The guillotine door was raised and the rat then moved across to the other side where it encountered an identical copy of object A and a different object B (Trial 1). After 1 min the sliding door was raised again to enable the rat to shuttle across where it found an identical copy of object B and object C (Trial 2). This continued for 20 trials using a total of 21 object pairs. In each trial the rat was allowed to collect one sucrose pellet from the food well. The position of the familiar object in relation to its previously viewed copy (same side, opposite side) was counterbalanced across each session. Furthermore, the order in which the objects were shown (either A to U or U to A) was counterbalanced across each rat group for each session.

Group Familiar saw the same 21 objects during every training session in a different order so that by the end of the training period the rats should be highly familiarised to this set of 21 objects. In the test session again the animals were presented with copies of the highly familiarised objects. Twenty-one different objects were used to match the sensory demands of the task.

The rats in group Novel received object exposure in the same way, i.e. rats were allowed to explore one novel object and one familiar object (familiar because it had been encountered at the other end of the maze in the preceding trial). For the first 6 training sessions, 21 different novel objects were used in every session. For the last 6 training sessions, these 126 objects were re-used but grouped into different sets of 21 objects and shown in a different order. In the test session group Novel saw the same 21 objects as group Familiar, hence all the conditions (objects seen, motor demands, length of exposure to the objects) were identical between the groups. As before, each test trial for group Novel comprised one novel object and one object encountered for a maximum of one minute in the previous trial.

On the last three training sessions, all rats were individually placed into a holding cage after each run for approximately 1 h to habituate them to this process for the test day. Group Control was habituated in the same way.

#### 2.2.4 Perirhinal cortex extraction

In the test session the rats were run on the task as described and then placed in a holding cage in a quiet and darkened room to minimise exposure to extraneous stimuli. One h after the end of the session rats were placed into an anaesthetic induction box filled with isoflurane until the heart stopped and guillotined. The brains were extracted, immediately frozen on dry ice and stored at −80 °C prior to dissection of the perirhinal cortex and RNA extraction. For group Control the same protocol was followed but without the prior behavioural test.

#### 2.2.5 Statistical analysis

Exploration of an object was defined as the rat directing its nose towards the object at a distance of <1 cm from the object. Sitting on the object or looking up while resting against the object was not counted as exploration. As a measure of behavioural performance, a discrimination ratio was calculated from the recorded object exploration (E) using the following formula:

$$Discrimination\;\;ratio=\frac{E_{novel}-E_{familiar}}{E_{total}}$$

For group Familiar (except for the first session), the E_novel_ and E_familiar_ refer to the less recently seen familiar object (last seen in the previous session) and the more recently seen familiar object (examined on the previous trial), respectively. The discrimination ratio was recalculated after every trial of a session (taking into account the exploration of all preceding trials of that session) yielding the updated discrimination ratio. Repeated-measures ANOVA was used to draw comparisons of discrimination between groups and sessions. Differences were followed up statistically using planned *t*‐tests. One sample *t*‐tests were used to determine if rats performed above chance (discrimination ratio of zero). For all statistical analyses a significance level of 0.05 was used.

### 2.3 RNA extraction

Caudal perirhinal cortex tissue was microdissected from frozen brains using the punch method. Brains were cut into 60 μm sections on the cryostat and sample corers of 0.5 mm inner diameter (Fine Science Tools, InterFocus, Linton, UK) were used to punch out the desired tissue area. Punches were taken bilaterally, 2 from each side, from 24 sections between −4.6 to −6.7 mm from bregma (Fig. 1c; [19]). Punched-out sections as well as map slices before and after samples were taken, were stained with 1% toluidine blue.

RNA was extracted from perirhinal cortex punches using TRIzol (Life Technologies) and the RNeasy Mini Kit (Qiagen, Hilden, Germany), following the RNA Clean-up protocol. RNA quality and integrity was measured on the 2100 Bioanalyzer (Agilent Technologies, Santa Clara, CA, USA) using the Eukaryote Total RNA Nano assay (Agilent Technologies) by the Bristol Genomics Facility.

### 2.4 RNA sequencing

Of the animals that were subjected to behavioural testing (*n* = 9 for both Novel and Familiar), five RNA samples per behaving group plus four Control samples were used for RNA sequencing, based on RNA concentration (>10 ng/μl), RNA integrity (RIN ≥ 7.0 and/or a 28S/18S ribosomal RNA (rRNA) ratio between 1.5 and 2.0) and on the rats’ behaviour in the test session of the bow-tie maze task. For the latter, group Novel rats were expected to show successful discrimination (discrimination ratio >0.2) and group Familiar rats were predicted not to show discrimination (discrimination ratio <0.2), based on the discrimination measures observed in the bow-tie maze task, previously, by Albasser et al. [14].

Library preparation and total RNA sequencing were performed by the Bristol Genomics Facility. Briefly, samples were selectively depleted of rRNA using the Low Input Ribominus Eukaryote System v2 and cDNA libraries were prepared from 250 ng total RNA using the Ion Total RNA-seq Kit v2 in combination with the Ion Xpress RNA-Seq Barcode 1–16 Kit (all kits from Life Technologies) by following the manufacturer’s protocol. Total RNA sequencing was performed on the Ion Proton System (Life Technologies). All of the samples were run twice, on two chips (chip type P1.1.17), producing single-end reads. The template and sequencing kits used were the Ion Proton One Touch 200 v3 Kit and the Ion Proton Sequencing 200 v3 Kit (both Life Technologies).

### 2.5 Data processing

The sequencing data was processed and analysed using the Galaxy platform [20], [21], [22]. The files were converted to FASTQ format using the FASTQ Groomer [23]. To assess the quality of the reads, the data were analysed via the FASTQC tool (http://www.bioinformatics.babraham. ac.uk/projects/fastqc/) with reference to the available guide on expected FASTQC results from Ion Proton sequencer data [24]. Alignment of the sequencing reads was performed via TopHat [25] using the rat reference genome Rnor_6.0 (GenBank Assembly ID GCA_000001895.4) submitted by the Rat Genome Sequencing Consortium. We used default TopHat parameters except for the range of expected intron lengths, which we set to 12–270,000 based on the current reference gene models. Unmapped reads were then re-aligned using Bowtie2 local alignments [26] and combined with the TopHat alignments. For each sample, the data from both chips were pooled before running the analysis pipeline. Reads were mapped to rat genes and the relative abundance of each transcript was quantified using the HTSeq package [27], counting only uniquely mapped reads and allowing for an alignment quality threshold of 10. Using these counts as input, differential gene expression analysis was performed using edgeR [28]. RNA sequencing data can suffer from noise from experimental sources unrelated to treatment groups. To mitigate this unwanted variation, we used RUVSeq [29] to adjust read counts before running edgeR on the adjusted counts. The raw and processed data files generated from the RNA sequencing have been deposited in NCBI’s Gene Expression Omnibus [30] and are accessible through *GEO: GSE84242* (http://www.ncbi.nlm.nih.gov/geo/query/acc.cgi? acc=GSE84242).

We used the SpliceGrapher package [31], [32] to assess alternative splicing (AS). From the reference models, we selected a single splice form to represent each gene, using the longest splice form as the canonical form for each gene. While this heuristic approach may omit some prevalent splice forms, using a consistent reference allows us to compare AS between samples at a coarse level. We ran SpliceGrapher using these canonical forms to predict AS activity represented in the RNA sequencing data and counted the number of AS events in each sample.

In addition to identifying those genes that appear to be up- or down-regulated between conditions, we also assessed possible changes in transcript expression. To this end we used iDiffIR, an analysis method designed to quantify changes in exon usage between two sets of RNA-Seq data (http://combi.cs.colostate.edu/idiffir/introduction.html). Exons called skipped exons or cassette exons may be excluded from some mRNA transcripts in a sample, hence the expression level for an individual exon may change relative to its gene’s overall expression. For cassette exons within a particular gene, iDiffIR detects changes in relative expression using RNA-Seq read counts to estimate relative exon and gene expression levels, and reports those exons whose relative expression levels appear to change significantly between samples. We ran iDiffIR with default settings (*q*-value multi-test correction with false discovery rate 0.05) and used the Rn6 version of the rat gene models to provide coordinates for all cassette exons. An adjusted *p*-value cut-off of 0.01 was used for comparisons.

### 2.6 Data analysis

Genes that had mean counts below 1 in any group were excluded from further analysis. Genes were considered differentially expressed genes (DEGs) if the read counts between two of the sample groups were significantly different at a significance level of *p* < 0.01 and if the up or downregulation was above a 25% threshold. In order to extract information regarding the types of genes that were differentially regulated, gene ontology analysis of DEGs based on enriched biological processes and pathways was performed using DAVID Bioinformatics Resources 6.7 [33], [34] utilising the GO_BP_FAT and KEGG pathway options. STRING 10 database [35] was used to predict high-confidence (0.700) protein interactions between DEGs. K-means clustering with a maximum of 6 clusters was performed for better visualisation of the interaction network. A list of transcription factors in the rat was downloaded from the curated database AnimalTFDB 2.0 [36] and compared with the detected DEGs.

## 3 Results

**Fig. 2:**
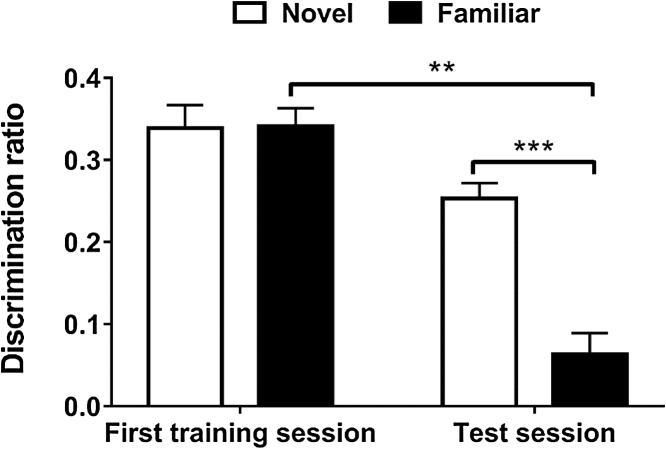
Discrimination in the bow-tie maze task. The updated discrimination ratio (±s.e.m.) achieved in the first training session and the test session of the bow-tie maze task is shown for rats that had been familiarised to the same set of objects over the course of the training (group Familiar) and for rats that explored novel objects in every session (group Novel). Only performance by those rats whose samples were selected for subsequent RNA sequencing is shown (*n* = 5 for both groups). Differences marked with asterisks are significant (**p < 0.01; ***p < 0.001).

Fig. 2 shows the mean discrimination ratio achieved in the first training session and the final test session of the bow-tie maze task. Rats belonging to group Familiar had been repeatedly exposed to the objects presented to them in the test session over 12 training sessions, therefore all the objects in the test session were highly familiar. Group Novel rats were exposed to the same set of objects in the test session, however to these rats all the objects were completely novel. Each object was encountered twice in the session, in consecutive trials, i.e. each trial consisted of an object that had not been seen in this session before and one that had just been encountered in the previous trial of the session. Rats belonging to group Novel showed significant discrimination between the novel objects they had never seen before and the novel objects encountered for the first time in the previous trial. In contrast, group Familiar rats showed a reduction in mean discrimination ratio in the test session, i.e. they did not discriminate between equally familiar objects − the highly familiar object last seen in the previous training session and the highly familiar object last seen in the previous trial of the test session. Statistical analysis using ANOVA detected a significant session by group interaction (*F*_1,8_ = 14.57, *p <* 0.01) as well as a significant main effect of session (*F*_1,8_ = 51.84, *p <* 0.0001) and of group (*F*_1,8_ = 16.78, *p <* 0.01). Post-hoc paired comparisons indicated that discrimination of group Familiar rats was reduced over the course of the training (First training session vs Test session; *F*_1,4_ = 65.35, *p* < 0.001) whereas no significant difference between discrimination in the first session and discrimination in the test session was observed for group Novel (*F*_1,4_ = 5.34, *p* > 0.05). Furthermore, there was a significant difference between both groups in the test session (*F*_1,8_ = 35.27, *p* < 0.0001) but not in the first session (*F*_1,4_ = 0.004, *p* > 0.05). To investigate whether the rats differentiated between the novel (or less recently seen highly familiar) object and the now familiar (or more recently seen highly familiar) object, the discrimination ratios were tested for their difference from zero discrimination. Rats belonging to group Novel showed discrimination that was significantly different from zero in both sessions (First training session, *t*_4_ = 11.89, *p <* 0.001; Test session, *t*_4_ = 13.60, *p <* 0.001). The discrimination ratio of group Familiar differed significantly from zero in the first session (*t*_4_ = 15.45, *p <* 0.001) and then gradually declined over the course of the experiment until it was no longer significant in session 11 (*t*_4_ = 2.60, *p* < 0.05), session 12 (*t*_4_ = 2.51, *p* < 0.05) and the test session (*t*_4_ = 2.42, *p* > 0.05) (Fig. 2, Supporting Fig. 1).

Analysis of cumulative exploration levels (Supporting Table 1) indicated a significant main effect of session (*F*_12,96_ = 9.829, *p <* 0.0001) and a significant main effect of group (*F*_1,8_ = 5.808, *p <* 0.05). Levels of exploration in the test session by group Familiar rats were significantly different compared to exploration in the first training session (*F*_1,4_ = 3.10, *p <* 0.05) and to exploration of group Novel rats in the test session (*F*_1,4_ = 5.53, *p <* 0.01). Supporting Table 2 shows cumulative discrimination values that have not been corrected for differences in exploration.

### 3.1 RNA sequencing raw data

RNA isolated from caudal perirhinal cortices selectively extracted from group Control, Novel and Familiar rats was subjected to RNA sequencing. Using the Ion Proton sequencer a total of 155 million reads were generated, which equates to 10 million reads per library. The mean read length was 83 nt with read lengths varying between 20 and 200 nt. 83% of bases had a quality score of ≥Q20, i.e. a 99% probability of having been correctly assigned. The sequence reads were aligned and mapped to the rat genome in a two-step process. Using TopHat an average of 7.18E + 06 reads (±2.02E + 06) per sample library mapped uniquely to 29,998 annotated genes. Re-alignment of unmapped reads using Bowtie2 produced a total of 9.98E + 06 (±3.25E + 06) average mapped reads per library. This corresponded to successful alignment of approximately 97% of total reads per library.

### 3.2 Differential gene expression

Using edgeR differential gene expression analysis, pairwise comparisons between the gene expression patterns of groups Novel, Familiar and Control were performed. Due to expected high variability between samples of tissue origin, RUVSeq analysis was first used to adjust counts depending on the expected variation (Supporting Fig. 2). The best separation between the samples of the two groups to be compared was consistently achieved when 4 sources of variation were assumed (*k* = 4), although complete separation was not achieved for the comparison between groups Novel and Familiar (Supporting Fig. 2c) and hence may exclude some genes that were differentially expressed.

**Fig. 3:**
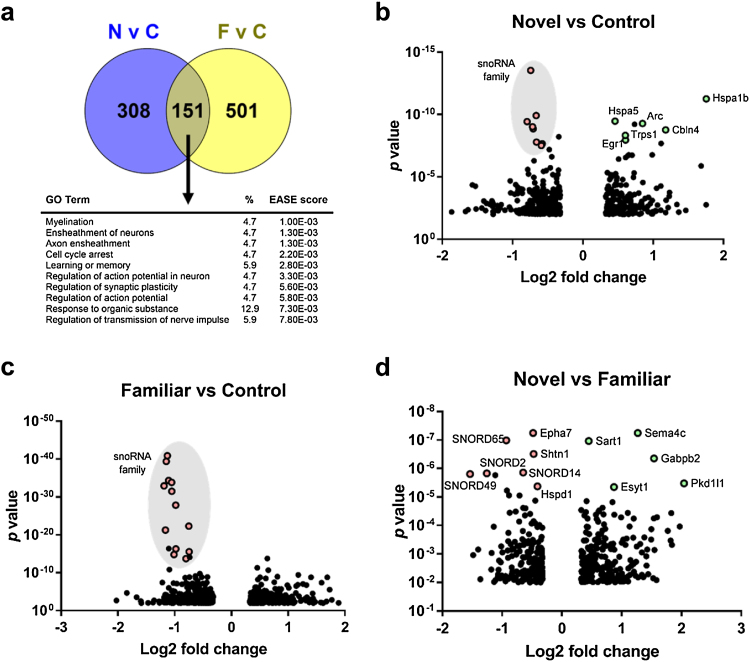
Distribution of DEGs. **a**, Overlap between DEGs in Novel and Familiar compared to Control and gene ontology analysis. **b**–**d**, Log2 fold changes versus *p* values are shown for significant genes. Highly significant genes or gene groups are highlighted.

DEGs were defined as those genes whose expression changed by at least 25% and that reached significance levels of *p <* 0.01 (Table 1, Fig. 3). The greatest number of DEGs (652) was found in the perirhinal cortex of group Familiar compared to Control, with 49% of genes being upregulated and 51% being downregulated genes. When group Novel was compared to Control, 459 DEGs were identified, with 39% being significantly upregulated and 61% significantly downregulated. Groups Novel and Familiar were found to have a proportion of the same genes activated, with 33% of those genes activated following exposure to novel stimuli (compared to naïve controls) also being differentially expressed following exposure to familiar stimuli (Fig. 3a).

**Table 1:** Number of DEGs detected by pairwise comparison of groups Novel, Familiar and Control. Total number of DEGs as well as up or downregulated DEGs are shown for *p* < 0.01 with a 25% minimum fold change.

	Total	Up	Down
Novel vs Control	459	188	271
Familiar vs Control	652	321	331
Familiar vs Novel	436	237	199

Analysis of highly significant DEGs showed a reduction in the levels of small nucleolar RNAs (snoRNAs) under both Novel and Familiar conditions, compared to Control (Fig. 3b,c). In group Novel, genes that were highly significantly upregulated compared to Control included transcription factors and immediate early genes such as *Arc* (*p* = 5.29E-10), *Egr1* (*p* = 1.11E-08) and *Trps1* (*p* = 5.65E-09) as well as heat shock proteins *Hspa1b* (*p* = 5.60E-12) and *Hspa5* (*p* = 3.44E-10). Genes upregulated with high significance in the Familiar group compared to the Novel group (Fig. 3d) were involved in transcriptional regulation and splicing − *Sart1* (*p* = 1.07E-07), *Gabpb2* (*p* = 4.37E-07) − and calcium signalling − *Pkd1l1* (*p* = 3.28E-06) and *Esyt1* (*p* = 4.44E-06). Both up and downregulated genes were found among those associated with cell–cell communication and neuron outgrowth, e.g. *Sema4c* (*p* = 5.62E-08), *Epha7* (*p* = 5.60E-08) and *Shtn1* (*p* = 3.06E-07).

### 3.3 Characterisation of differentially expressed genes

**Fig. 4:**
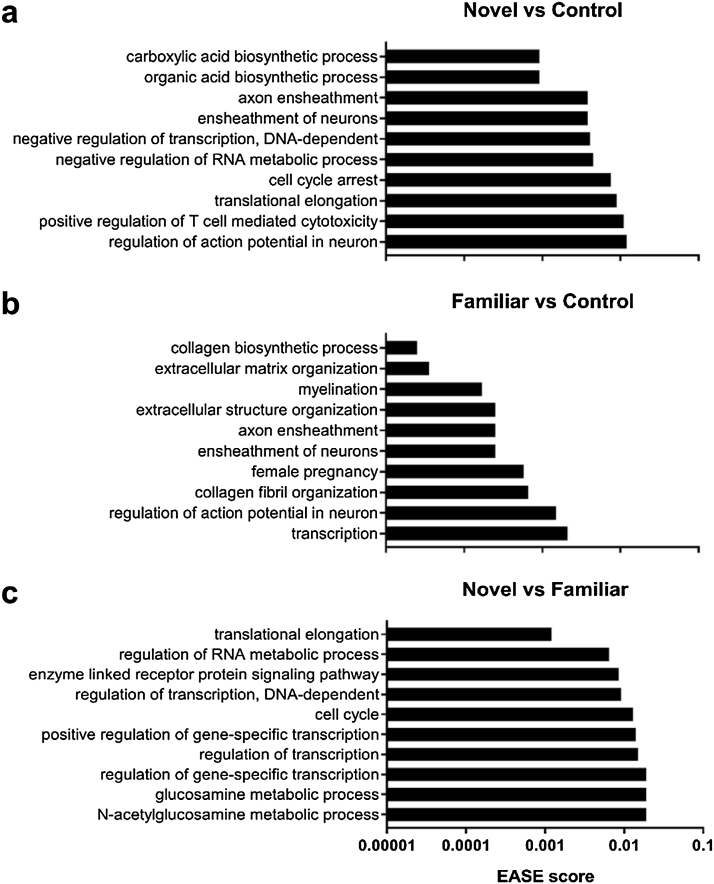
Gene ontology analysis. Top 10 significantly (EASE score < 0.05) enriched gene ontology terms amongst DEGs from each comparison are shown.

Regulation of transcription and translation as well as action potential regulation were among the gene ontology terms significantly enriched among DEGs found in group Novel compared to Control or group Familiar (Fig. 4a,c). DEGs in group Familiar compared to Control were enriched in extracellular matrix and structure organisation, as well as genes relating to neuronal action potentials and transcription (Fig. 4b). Pathway analysis showed enrichment of genes involved in ribosome pathways when group Novel was compared to Control (*p* = 1.30E-03) or Familiar (*p* = 2.10E-04) while significant pathways for group Familiar compared to Control were extracellular matrix receptor interaction (*p* = 1.30E-03) and focal adhesion (*p* = 1.80E-02).

**Fig. 5:**
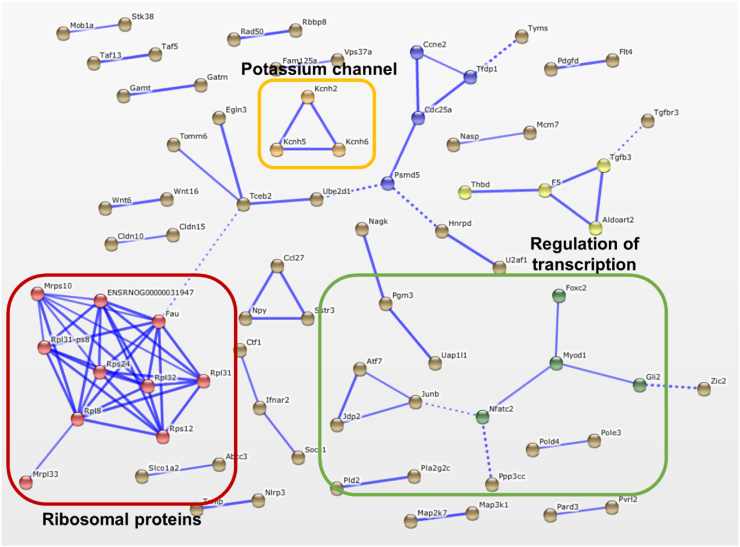
Predicted gene interactions. Interactions between the DEGs detected between groups Familiar and Novel. Thicker lines represent stronger associations. Different colours denote different interaction clusters.

Potential and confirmed interactions between DEGs were visualised using the STRING tool (Fig. 5, Supporting Figs. 3,4). For all the comparison between groups Novel, Familiar and Control, clusters of DEGs were found associated with transcription, translation and protein processing. For the Familiar vs Novel comparison a small cluster of potassium channel-related genes was also detected. DEGs upregulated in Familiar compared to Control showed further interactions between genes coding for extracellular matrix proteins.

### 3.4 Differential expression of transcription factors and immediate early genes

**Fig. 6:**
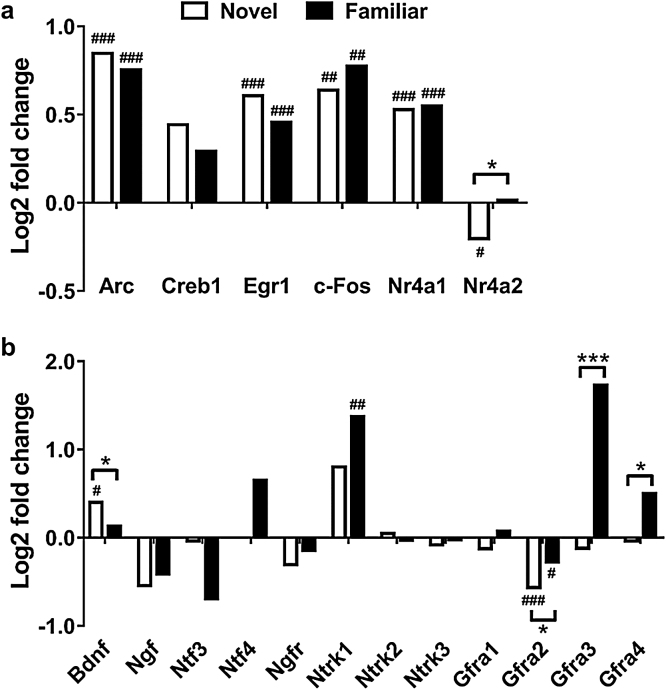
Differential gene expression of memory-related genes. Log2 fold changes of transcripts associated with signalling pathways linked to recognition memory are shown for samples from Novel (*n* = 5) or Familiar (*n* = 5) perirhinal cortices, compared to Control levels (*n* = 4). **a**, Transcription factors and immediate early genes. **b**, Neutrophic signalling genes. Log2 fold changes were significant for ^#^*p* < 0.05; ^##^*p* < 0.01; ^###^*p* < 0.001. * indicates significant difference between transcript levels of groups Novel and Familiar for *p < 0.05, ***p < 0.001.

The RNA sequencing data was further analysed to examine the effects of exposure to novel or familiar objects on the expression levels of transcription factors and immediate early genes associated with recognition memory processes in the perirhinal cortex [5], [6], [7], [8], [9], [10], [11]. For most of these transcription factors an increase in gene expression was observed in group Novel compared to group Familiar or Control (Fig. 6a). For group Novel a significant and approximately 1.5–2.0-fold upregulation compared to the Control group was observed for *Arc*, *Egr1*, *c-Fos* and *Nr4a1* (*Arc*, *p* = 5.29E-10; *Egr1*, *p* = 1.11E-08; *c-Fos*, *p* = 8.13E-03; *Nr4a2*, *p* = 3.05E-02). Creb1 transcripts showed a 36% increase which was not significant (*p* = 7.34E-02), while Nr4a2 was significantly downregulated in group Novel (*p* = 9.65E-04).

A similar increase in gene expression for *Arc*, *Egr1, c-Fos* and *Nr4a1 was* observed in group Familiar (*Arc*, *p* = 2.98E-08; *Egr1*, *p* = 3.75E-05; *c-Fos*, *p* = 1.78E-03; *Nr4a1*, *p* = 4.74E-04). Group Familiar also showed a small non-significant increase in *Creb1* transcript levels (*p* = 2.79E-01). Levels of *Nr4a2* mRNA however were unchanged in comparison with Control (*p* = 8.88E-01).

Analysis of differential expression between groups Novel and Familiar of transcription factors that have been previously associated with recognition memory formation showed that only Nr4a2 transcript levels were significantly altered between the two groups, with higher expression detected in group Familiar (*p* = 1.52E-02). However, comparison with a database of rat transcription factors showed differential gene expression between Novel and Familiar for a number of additional transcription factors (Table 2); zinc finger proteins in particular were seen to be downregulated in group Familiar compared to Novel.

**Table 2:** Transcription factors with significantly different transcript levels between Novel and Familiar. For the comparison between the perirhinal transcriptomes of groups Novel and Familiar the DEGs that are transcription factors are listed, including the family of transcription factors they are classed in. bHLH, basic helix-loop-helix; bZIP, basic leucine zipper; C/EBP, CCAAT-enhancer-binding protein; E2F, E2 factor; PR, progesterone receptor; RHD, Rel homology domain; ZBTB, zinc finger and BTB domain containing; zf, zinc finger.

	Ensembl ID	Name	Family	*p* value	Log2 FC
↑	ENSRNOG00000023433	Gata6	zf-GATA	<0.001	1.97
	ENSRNOG00000011306	Myod1	bHLH	<0.001	1.34
	ENSRNOG00000012175	Nfatc2	RHD	0.001	0.96
	ENSRNOG00000007261	Gli2	zf-C2H2	0.005	0.78
	ENSRNOG00000010918	Cebpa	C/EBP	<0.001	0.70
	ENSRNOG00000015269	Atf7	C/EBP	0.003	0.53
	ENSRNOG00000006831	Pgr	PR	0.004	0.50
	ENSRNOG00000042838	Junb	TF_bZIP	<0.001	0.46
	ENSRNOG00000000974	Zfp358	zf-C2H2	0.002	0.45
	ENSRNOG00000008224	Jdp2	TF_bZIP	0.006	0.40

↓	ENSRNOG00000019222	Tfdp1	E2F	0.004	−0.33
	ENSRNOG00000002163	Klf3	zf-C2H2	0.005	−0.33
	ENSRNOG00000015925	Zfp131	ZBTB	0.003	−0.33
	ENSRNOG00000017863	Zeb1	Homeobox	<0.001	−0.34
	ENSRNOG00000016971	Znf612	zf-C2H2	<0.001	−0.35
	ENSRNOG00000001099	Rbak	zf-C2H2	0.001	−0.47
	ENSRNOG00000020762	Zfp260	zf-C2H2	0.004	−0.52
	ENSRNOG00000030416	Zfp870	zf-C2H2	0.001	−0.75
	ENSRNOG00000022391	Znf711	zf-C2H2	0.004	−0.76
	ENSRNOG00000017986	Zfp458	zf-C2H2	0.001	−0.77

### 3.5 Differential expression of neurotrophic signalling genes

Fig. 6b shows the changes in levels of transcripts associated with neurotrophic signalling genes. For group Novel a significant upregulation of *Bdnf* transcripts (*p* = 1.08E-02) and a downregulation of transcripts for GDNFα family receptor *Gfra2* (*p* = 4.01E-05) were observed. In the perirhinal cortex of group Familiar, receptors *Ntrk1* (*p* = 6.60E-03) and *Gfra3* (*p* = 2.65E-06) were upregulated while *Gfra2* was downregulated (*p* = 2.90E-02).

When groups Novel and Familiar were compared, significant differences in the levels of *Bdnf* and of GNDF receptors were observed. *Bdnf* mRNA levels were higher in the Novel condition (*p* = 4.31E-02). In contrast, transcripts levels of *Gfra2* (*p* = 6.90E-03), *Gfra3* (*p* = 3.64E-05) and *Gfra4* (*p* = 3.71E-02) were higher in group Familiar.

### 3.6 Alternative splicing

**Fig. 7:**
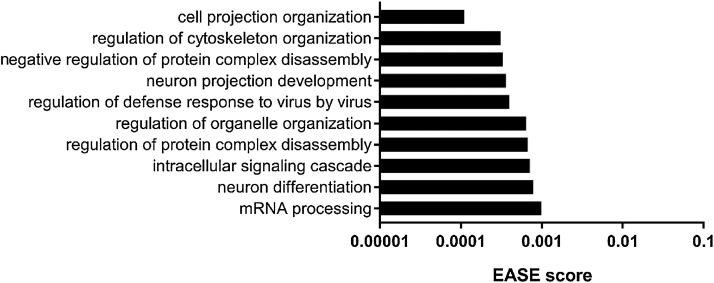
Gene ontology analysis of differentially alternatively spliced genes. Top 10 significantly (EASE score < 0.05) enriched gene ontology terms amongst genes whose transcripts show differences in alternative splicing events between groups Novel and Familiar.

Genes coding for proteins involved in splicing were found to be significantly altered after exposure to novel or familiar objects and we therefore interrogated our data for alternative splicing events (Table 3). The number of total alternative splicing events was increased in the two experimental groups compared to Control, with the highest occurrence of alternative splicing detected in group Familiar. In all groups the predominant alternative splicing event was exon skipping. The largest relative increase in alternative splicing events in groups Novel and Familiar compared to Control was caused by intron retention, which was increased by over 30% in both groups. While the number of genes whose transcripts underwent alternative splicing was also increased in Novel or Familiar compared to Control, no difference in the number of alternatively spliced genes was detected between groups Novel and Familiar. Examined over all types of alternative splicing, 508 genes showed differences in the number of alternative splicing events of their transcripts between groups Novel and Familiar. This was predominantly due to changes in the number of exon skipping events (262 genes). For 8 of these genes there was a significant difference in exon skipping events between Novel and Familiar (Table 4). Furthermore, a significant enrichment of gene ontology terms related to the regulation of the cytoskeleton, cell projection and mRNA processing was observed for those genes whose transcripts showed altered alternative splicing when groups Novel and Familiar where compared (Fig. 7).

**Table 3:** Alternative splicing events. The numbers of total alternative splicing events as well as of the individual alternative splicing events detected for each rat group are shown (‘Events’). In addition, the number of genes whose transcripts underwent alternative splicing is given (‘Genes’).

	Control		Novel		Familiar	
	Events	Genes	Events	Genes	Events	Genes
Alternative donor	374	122	393	138	453	132
Alternative acceptor	432	156	435	184	505	186
Exon skipping	578	365	677	401	718	392
Intron retention	138	132	184	178	189	179
*Total*	*1522*	*645*	*1689*	*738*	*1865*	*724*

**Table 4:** Differentially spliced genes. Genes with significant differential alternative splicing between groups Novel and Familiar, based on exon-skipping events. *p* values were adjusted for multiple comparisons.

Ensembl ID	Name	Exon coordinates	*p* value	Log2 FC
ENSRNOG00000029450	Adgrb1	7:56754-56793	0.000000	−0.90
ENSRNOG00000029450	Adgrb1	7:56754-56808	0.000000	−0.88
ENSRNOG00000029450	Adgrb1	7:18472-18615	0.000175	−0.74
ENSRNOG00000029450	Adgrb1	7:11557-11730	0.015876	−0.84
ENSRNOG00000060518	LOC257642	14:536-647	0.000000	1.47
ENSRNOG00000015396	Sptan1	3:60928-60945	0.000017	1.59
ENSRNOG00000004067	Nrcam	6:50009-50044	0.000269	1.10
ENSRNOG00000010048	Dctn1	4:6613-6630	0.005025	2.14
ENSRNOG00000002823	Mapk9	10:29129-29200	0.010132	1.50
ENSRNOG00000012482	Ndrg4	19:2503-2541	0.010698	0.65
ENSRNOG00000022802	Tmem184b	7:2964-2954	0.040522	−1.69

## 4 Discussion

Using deep sequencing we examined the perirhinal cortex transcriptome 1 h after a recognition memory task, in which rats were exposed to novel objects or to objects which had been repeatedly presented so as to become familiar. The bow-tie maze task was used as it enables recognition memory to be tested over multiple trials. Rats exposed to highly familiar objects in the test session explored significantly less overall than rats exposed to novel objects, as they had become habituated to the familiar objects. The discrimination ratio was calculated to compensate for these differences in total exploration. As has been observed previously [14], rats in group Novel showed significant discrimination between the novel objects and the objects encountered on the previous trial, while rats in group Familiar did not discriminate between familiar object pairs at the end of the training. Analysis of behaviour in group Familiar over all training session, showed a gradual drop in discrimination values. While group Familiar rats were initially able to discriminate between the less recently seen (last seen in the previous session) and the more recently seen (last seen in the previous trial of the current session) object, they no longer showed recency discrimination toward the end of the training and in the test session, likely due to the high familiarity of the presented objects.

### 4.1 Detection of differences in the transcriptome of rats exposed to novel or familiar objects

The present study sought to examine the transcriptome of recognition memory in rats. The bow-tie maze procedure enables separate groups of rats to be exposed to novel or familiar objects within the same apparatus, with matched exploration patters and food reward. Thus the differential expression of DEGs identified in groups Novel and Familiar in this study is likely to be related to the relative familiarity of the objects and not to nonspecific factors such as levels of attention or locomotor activity. This conclusion is supported by the observation that: (i) there is a significant upregulation of several transcription factors previously shown to be involved in recognition memory function [6], [7]; (ii) the only difference between groups Novel and Familiar were the objects they were exposed to (the behavioural training remained the same) and different DEGs between the behaving groups were identified.

Previous studies that compared gene expression in perirhinal cortices of rats exposed to either novel or familiar 3D objects or 2D images found increases in protein levels upon neuronal activation in response to the novel compared to the familiar stimulus [10], [11], [12], [13], [14], [37], [38]. Intriguingly, when the transcriptomes of groups Novel and Familiar were compared, a higher proportion of genes was found to be upregulated in group Familiar compared to Novel. At first glance, this finding appears to contrast with the observation that familiarity discrimination is mediated by neuronal response reduction in the perirhinal cortex when a stimulus is re-encountered [39]. However, it has been argued that these response reductions are mediated by a long-term depression-like mechanism in the perirhinal cortex [40], [41], the induction and maintenance of which will necessitate long-term changes in gene expression profiles. Further it has recently been reported that there are differences in the neuronal responses to 3D object stimuli presented in an arena compared to 2D computer-generated stimuli presented in the paired-viewing task [42]. It has been argued that these differences may reflect the fact that a high proportion of perirhinal neurons are devoted to processing familiarity information, only a proportion of which alter their response depending on the novelty or familiarity of the stimuli. In addition, all the objects presented to group Familiar had been previously encountered at different times and as the perirhinal cortex has been shown to encode information about the temporal context of an object presentation [11], [43], activation in response to a familiar object may reflect object recency information. Alternatively, the higher gene expression levels in group Familiar may reflect reconsolidation processes, i.e. the reactivation of the memory trace in response to re-exposure to the familiar stimuli, with subsequent new protein synthesis to ensure that the memory trace remains in long-term memory. Reconsolidation processes have been shown to correlate with an increased expression of certain genes [44], [45], [46], similarly to consolidation of memory, which we expect to occur in the group Novel perirhinal cortices. A reconsolidation of recognition memory traces in the perirhinal cortex of group Familiar rats would be in agreement with the observed lack of reduction of transcriptional activity in the Familiar condition, compared to Control and Novel.

### 4.2 Effects on transcription and translation-related genes

In the present study, gene ontology, pathway and protein interaction analyses as well as the examination of highly significant genes provided consistent results and together suggest that genes controlling transcription, translation and RNA processing were activated following exposure to both novel and familiar stimuli, compared to Control, while genes controlling transcription, translation, splicing and cell–cell communication were differentially activated between groups Novel and Familiar.

The observation that transcription and translation-related processes were enriched among detected DEGs correlates with previous findings that both transcription and translation are required for recognition memory function and that an increase in levels of transcription factors and immediate early genes occurs during consolidation of recognition memory traces [4], [11], [12], [13]. It also supports the possibility that memory reconsolidation processes occur in the perirhinal cortex in response to the re-presentation of familiar stimuli, as reconsolidation also requires protein synthesis and necessitates the involvement of transcription factors and immediate early genes [44], [45].

Increases or activation of transcription factors and immediate early genes c-Fos [5], [12], [11], [12], [13], CREB [10], Egr1 [5], [7], Arc [6] and Nr4a2 [8] in the perirhinal cortex have previously been associated with recognition memory. In line with these findings, we observed a significant increase in the transcript levels of *c-Fos*, *Egr1* and *Arc* in both groups of rats undergoing the recognition memory task. *Creb1* was also increased in both groups but this did not reach significance, however, previous studies measured levels of activated phosphorylated CREB protein [10] which may not be driven by an increase in the level of *Creb1* transcripts.

While transcription factor Nr4a2 was previously found to be upregulated after an object recognition memory task [8], transcriptomic analysis of the perirhinal cortex in the present study showed a downregulation of *Nr4a2* expression following exposure to novel objects compared to familiar objects and the Control group. In contrast, *Nr4a1* a second member of the Nr4 nuclear receptor family, which has previously been associated with object location but not object recognition memory [8], was found to be significantly upregulated in groups Novel and Familiar compared to Control. The lack of agreement with previous findings may be related to differences in quantification, as the earlier study measured Nr4 nuclear receptor expression level at protein level, rather than measuring transcripts. Therefore, further validation and investigation of these two transcription factors may be necessary.

The possibility cannot be excluded that the up- or downregulation of those genes that were significantly altered in *both* Novel and Familiar, compared to Control levels, could be due to the rats undergoing an active task rather than because they were specifically exposed to novel or familiar stimuli. The advantage of the bow-tie maze approach lies in the possibility of investigating differences between rats consolidating novel memory traces and those exposed to familiar objects or reconsolidating familiar memory traces. While both Novel and Familiar groups showed increases in transcription factors at mRNA levels in the perirhinal cortex, it is of particular interest to investigate differences between the two groups. Comparison with a database of known transcription factors highlighted 20 further transcription factors that were differentially expressed between the two groups and warrant further investigation.

### 4.3 Effects on neurotrophic signalling genes

In line with previous studies [17], [18], there was a significant increase in levels of *Bdnf* in group Novel compared to Familiar and Control. Additionally, we observed that GDNF family receptors were differentially expressed at mRNA level between rats exposed to novel objects and rats that explored familiar objects. Previous studies have shown that components of the neurotrophic signalling pathway may be important for memory function [15], [16], [17], [18]. Neurotrophic tyrosine kinase receptors in the cell membrane activate downstream signalling pathways such as the ERK/MAPK pathway which is required for recognition memory [7], [15], [47] and these receptors are activated by neurotrophins including BDNF and GDNF. As the four GDNFα receptor subtypes vary in their affinity to different neurotrophic ligands [48], it is possible that the sensitivity of perirhinal neurons to different GDNF family ligands may be regulated in response to novel or familiar stimuli. Our data suggests that the role of the neurotrophic signalling pathway in recognition memory merits further investigation.

### 4.4 Effects on extracellular matrix genes

A further class of genes that was found to be enriched among DEGs in the Familiar perirhinal cortex compared to Control and Novel were genes associated with the extracellular matrix which were found to be both up and downregulated. Perineuronal nets, a layer of condensed extracellular matrix found around soma and proximal dendrites of neuronal subpopulations, have been shown to be important for the regulation of synapse formation and adult plasticity in the central nervous system (reviewed in [49]. In the perirhinal cortex, depletion of perineuronal nets has been shown to improve recognition memory function and to facilitate perirhinal long-term depression [50]. Our data support the hypothesis that the extracellular matrix plays an important role in recognition memory and suggests that investigating genes and proteins involved in extracellular matrix and perineuronal nets within the perirhinal cortex may further our understanding of the molecular processes underlying recognition memory.

### 4.5 Effects on alternative splicing

Genes related to alternative splicing were enriched among the DEGs. These included snoRNAs that, in addition to their role in RNA modification (reviewed in [51]), have also been shown to modulate alternative splicing [52]. We investigated whether there were any changes in alternative splicing among the detected transcripts. There was a distinct increase in the number of alternative splicing events and alternatively spliced genes in the animals undergoing the recognition memory task. While there was an increase in the total number of splicing events in the perirhinal cortex of group Familiar compared to Novel, the number of alternatively spliced genes hardly changed. A possible explanation for this observation is that the large increase in alternative splicing events in group Familiar is caused by additional alternative splicing events on those transcripts that also undergo some alternative splicing in the Novel condition.

Exon skipping was identified as an alternative splicing event which showed significant differences between groups Novel and Familiar. Of those genes that showed significant differential splicing, adhesion G protein-coupled receptor B1 (Adgrb1), Ngcam-related cell-adhesion molecule (Nrcam) and N-myc downstream-regulated gene 4 (Ndrg4) have been shown to be required for hippocampus-dependent spatial learning and memory [53], [54], [55]. Both Adgrb1 and Nrcam are located at the synapse and are thought to play a role in synapse formation and function [56], [57], [58]. Adgrb1 as well as Ndrg4 regulate the ERK/MAPK signalling cascade [58], [59], which has been implicated in recognition memory function in the perirhinal cortex [47]. Moreover, *Mapk9* exon usage was also found to be significantly different between Novel and Familiar.

Alternative splicing has been shown to be a critical post-transcriptional process in the nervous system [60]. By increasing functional diversity of synaptic proteins, alternative splicing could contribute to the formation and plasticity of synapses, allowing them fulfil very specific function whilst retaining their diverse properties. Despite its potential significance, the role of alternative splicing in recognition memory has not been explored so far. Taken together our results suggest that alternative splicing may mediate the expression of RNA transcripts important for recognition memory consolidation and potentially reconsolidation processes in the perirhinal cortex.

## 5 Conclusion

In summary our data revealed a multitude of gene expression differences in the rat perirhinal cortices following a recognition memory task, including differences in genes that have thus far not been explored in the context of recognition memory. With regard to the hypotheses defined at the start of the study we have found firstly that transcription factors are significantly altered following exposure to novel objects as well as following exposure to familiar objects. Secondly, neurotrophic signalling pathway genes are differentially altered in the perirhinal cortex of rats that have explored novel or familiar objects and we have identified GDNF family receptors as potential genes of interest. Thirdly, we observed that alternative splicing occurred in the perirhinal cortex and may therefore contribute to regulatory processes underlying recognition memory consolidation. In addition, genes coding for proteins that form the extracellular matrix were identified as differentially expressed, which points towards a potential role of this dynamic structure in perirhinal recognition memory consolidation.

While not all genes previously associated with recognition memory were found to be differentially expressed, it is important to note that this study produced a snapshot of the transcriptome of the caudal perirhinal cortex at a single time point after the memory task. Analysis of further time points may provide a better understanding of the genes involved in the (re-)consolidation processes that occur after exposure to novel (or familiar) objects and which thus mediate recognition memory formation. New more selective techniques, such as translating ribosome affinity purification (TRAP) [61], could be used to refine which transcripts are analysed, firstly by examining only those transcripts that at that instant are being translated, and secondly by targeting specific cell types within a tissue.

To conclude, this study provides a proof of concept that analysing the transcriptome after a behavioural task using deep sequencing can provide new insights and may be an enriching tool to investigate novel potential target genes.

## Conflict of interest

The authors declare no competing financial interests.
