# Supplementary material for: Recognition memory-induced gene expression in the perirhinal cortex: A transcriptomic analysis
Source: Behav Brain Res. 2017 Jun 15;328:1–12. doi: 10.1016/j.bbr.2017.04.007 (PMC5469443; doi:10.1016/j.bbr.2017.04.007)
Supplement: Supplementary file 1 [file mmc1.docx]

**Recognition memory-induced gene expression in the perirhinal cortex: a transcriptomic analysis.**

Scott, H., Rogers, M.F., Scott, H.L., Campbell, C., Warburton, E.C. and Uney, J.B.

SUPPORTING INFORMATION

Figure legends

**Supporting Figure 1 – Recency discrimination of group Familiar rats.** Discrimination ratios of group Familiar rats are shown as means ± s.e.m. over all 12 training sessions and the final test session. Apart from the first session, in which all objects were novel to the rats, the discrimination ratio reflects recency discrimination between two familiar objects, one last seen on the previous trial, the other last seen in the previous session. Significance from a discrimination ratio of zero is shown: ^#^*p* < 0.05, ^##^*p* < 0.01, ^#^*p* < 0.001.

**Supporting Figure 2 – Analysis of relative log-expression (RLE) and principal components analysis (PCA) for gene expression comparisons.** The high variability in the raw data appears to be addressed after normalisation using RUVSeq (upper). PCA plots (lower) show that separation is achieved between the groups when we use a general linear model (GLM) with *k* = 4 sources of variation, except for Novel vs Familiar comparison where clear separation is not achieved between the groups after normalisation. **a**, Novel vs Control, **b**, Familiar vs Control, **c**, Familiar vs Novel.

**Supporting Figure 3 – Predicted interactions between DEGs from Novel versus Control comparison.** Thicker lines represent stronger associations. Different colours denote different interaction clusters.

**Supporting Figure 4 – Predicted interactions between DEGs from Familiar versus Control comparison.** Thicker lines represent stronger associations. Different colours denote different interaction clusters.

Supporting Figures





**Supporting Figure 1**


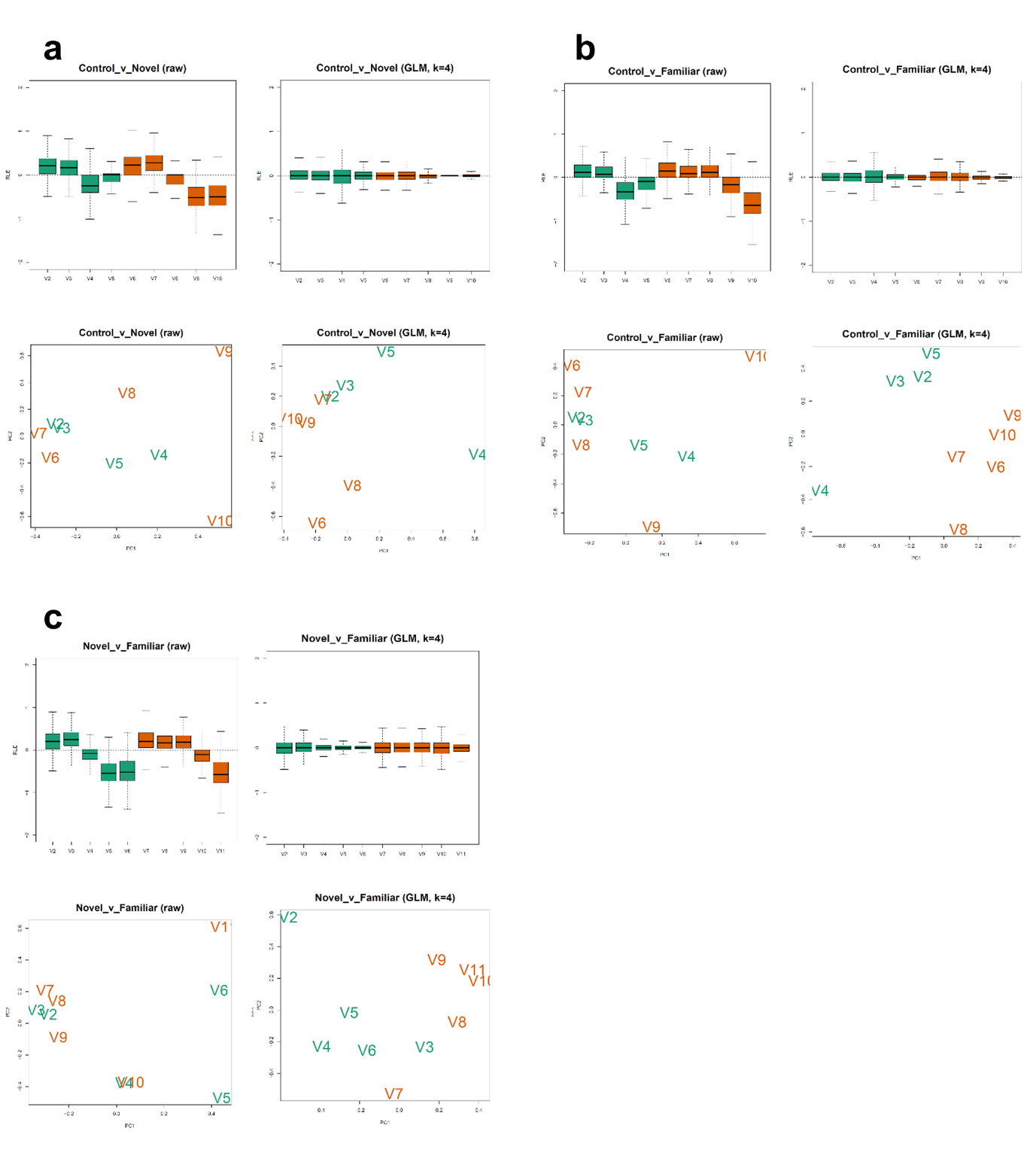


**Supporting Figure 2**


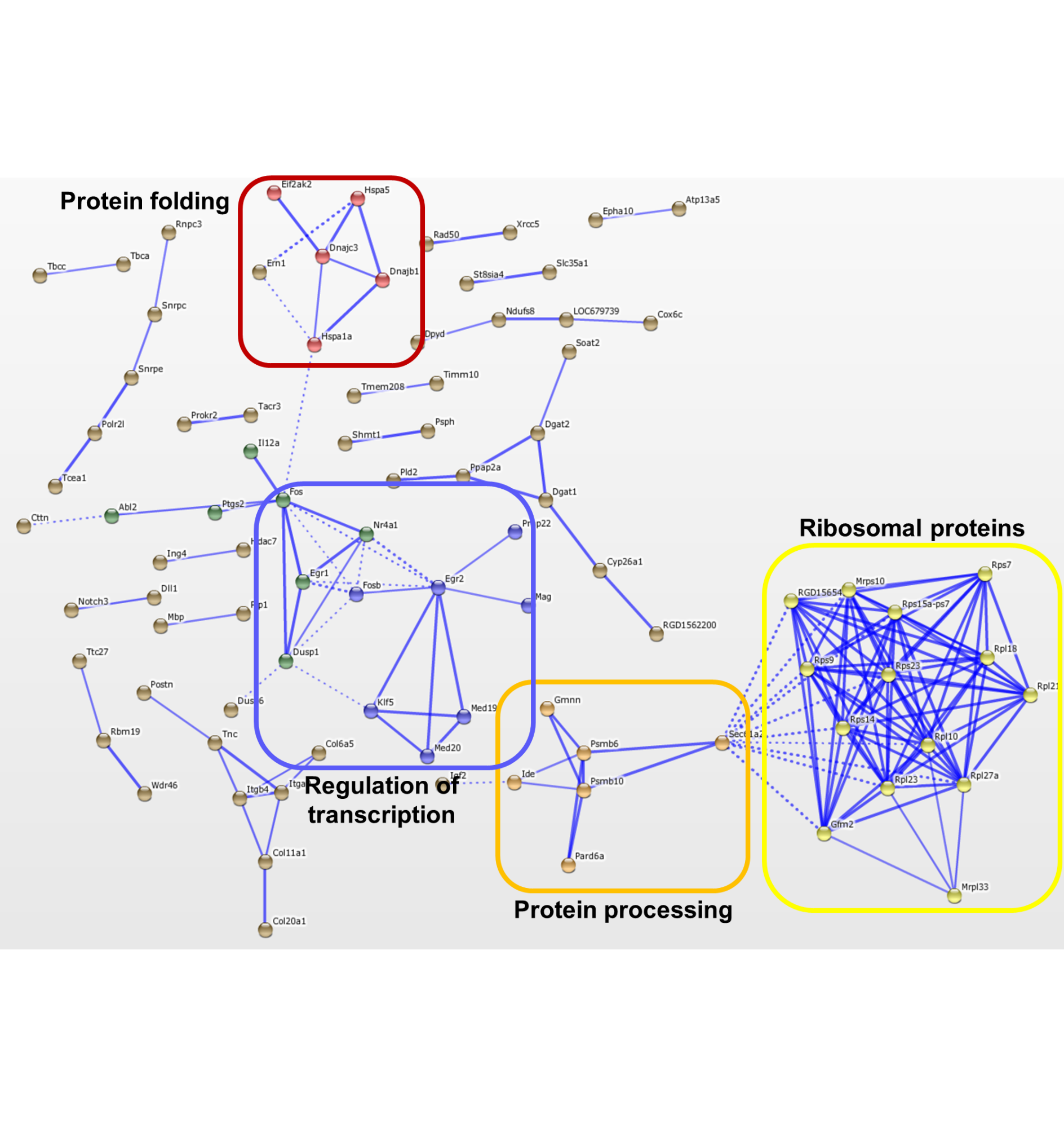


**Supporting Figure 3**


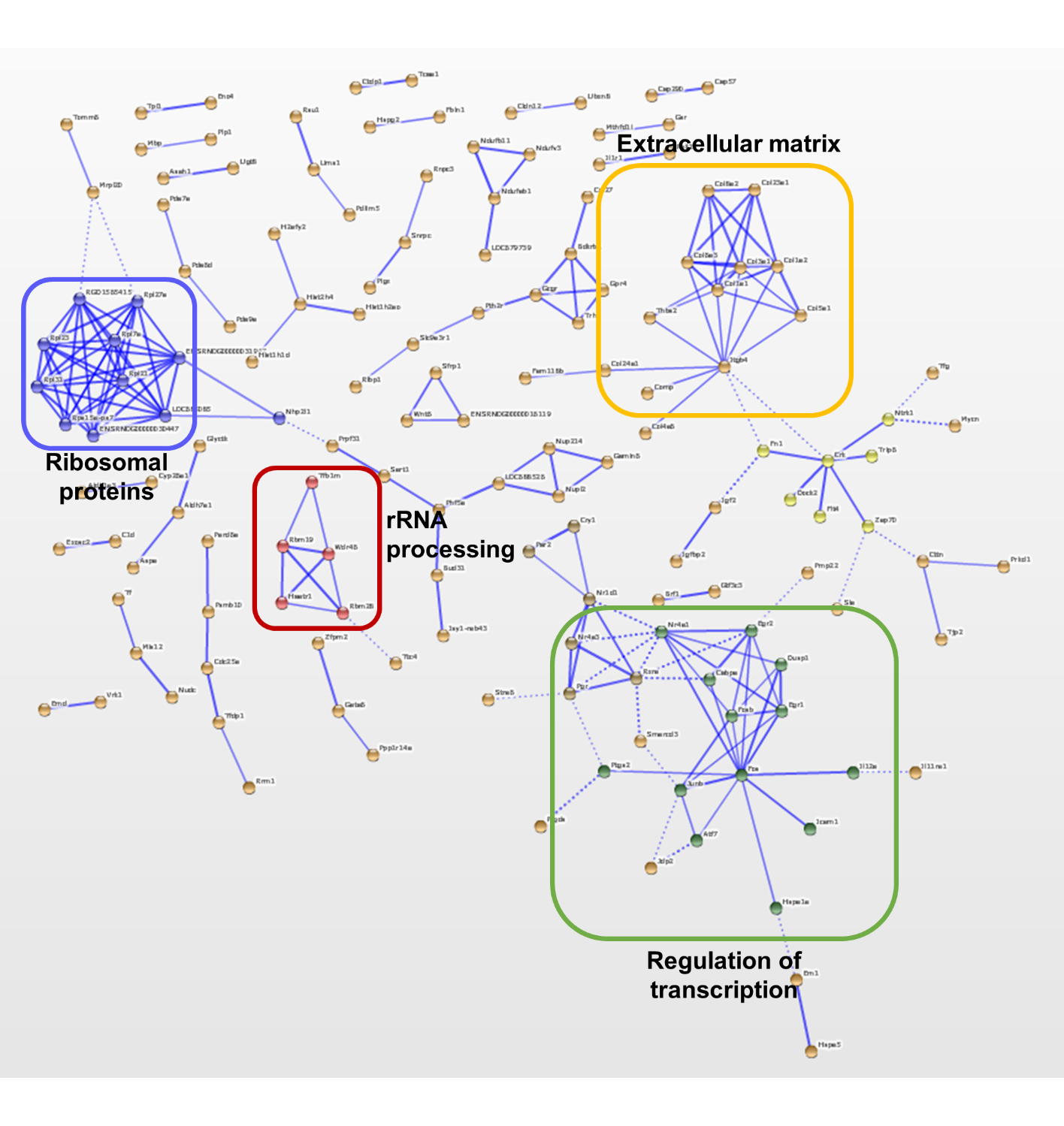


**Supporting Figure 4**

Supporting Tables

**Supporting Table 1 – Cumulative exploration in the bow-tie maze task.** Cumulative exploration of group Novel and group Familiar over all trials during the training sessions and in the test session are presented as means (± s.e.m.). Significant differences in exploration between the two groups are indicated; **p* < 0.05, ***p* < 0.01.

| **Session** | **Group Novel** | **Group Familiar** |
| --- | --- | --- |
| **1** | 294.74 (±18.49) | 321.19 (±12.87) |
| **2** | 253.04 (±17.87) | 217.62 (±18.72) |
| **3** | 217.72 (±24.00) | 192.73 (±19.95) |
| **4** | 193.19 (±29.84) | 175.68 (±24.29) |
| **5** | 269.79 (±16.99) | 237.54 (±27.66) |
| **6*** | 247.45 (±14.92) | 179.02 (±13.83) |
| **7** | 222.44 (±13.43) | 200.64 (±17.42) |
| **8** | 164.35 (±6.65) | 176.27 (±20.15) |
| **9** | 202.20 (±15.74) | 174.18 (±20.90) |
| **10** | 159.19 (±7.06) | 155.28 (±8.25) |
| **11** | 171.30 (±13.49) | 151.23 (±11.85) |
| **12*** | 217.87 (±21.33) | 152.24 (±13.10) |
| **Test**** | 266.79 (±8.61) | 196.12 (±15.76) |

**Supporting Table 2 – Cumulative discrimination in the bow-tie maze task.** Cumulative discrimination, i.e. difference in exploration between the two presented objects, of group Novel and group Familiar over all trials during the training sessions and in the test session are presented as means (± s.e.m.). Significant differences in exploration between the two groups are indicated; **p* < 0.05, ****p* < 0.001.

| **Session** | **Group Novel** | **Group Familiar** |
| --- | --- | --- |
| **1** | 100.70 (±13.30) | 110.21 (±10.82) |
| **2** | 78.32 (±15.17) | 59.68 (±10.50) |
| **3** | 55.41 (±11.58) | 26.39 (±11.28) |
| **4** | 68.04 (±14.09) | 37.73 (±9.49) |
| **5** | 65.96 (±12.09) | 37.08 (±8.06) |
| **6** | 49.80 (±14.07) | 19.88 (±6.58) |
| **7** | 34.86 (±8.84) | 20.57 (±9.93) |
| **8** | 40.13 (±7.87) | 33.06 (±10.06) |
| **9*** | 44.68 (±9.17) | 18.64 (±1.83) |
| **10** | 26.70 (±7.69) | 21.92 (±3.00) |
| **11** | 47.74 (±12.47) | 17.92 (±7.26) |
| **12*** | 45.61 (±7.64) | 15.03 (±7.75) |
| **Test***** | 68.17 (±7.30) | 12.84 (±5.07) |
